# The National Educational Panel Study (NEPS)—framework, design, and research potential

**DOI:** 10.1007/s11618-023-01156-w

**Published:** 2023-06-01

**Authors:** Cordula Artelt, Michaela Sixt

**Affiliations:** 1 Leibniz-Institut für Bildungsverläufe, Wilhelmsplatz 3, 96047 Bamberg, Germany; 2 Lehrstuhl für Bildungsforschung im Längsschnitt, Otto-Friedrich-Universität Bamberg, Wilhelmsplatz 3, 96047 Bamberg, Germany

## Setting up a national educational panel study

Initiated and funded by the Federal Ministry of Education and Research, in 2008 an interdisciplinary network led by H.-P. Blossfeld as PI^1^ was established to set up and implement the National Educational Panel Study (NEPS). The goal was to provide data for the description and explanation of individual competence development and educational trajectories over the entire life course in Germany. From the outset NEPS data were made available (free of charge) to the scientific community for research purposes. Furthermore, data collection aimed at educational reporting and thus at enabling evidence-based policy in the fields of (early) education, labor market participation, and lifelong learning.

The need to establish a National Educational Panel Study arose, among other things, from the fact that there are no register-based data across the federal states in Germany that can provide longitudinal information on educational levels and trajectories. More than ten years after the start of the National Educational Panel Study, the situation regarding (the use of) register data in educational research has not changed significantly (c.f. Artelt et al. 2019), although initiatives and statistical solutions to establish a “Bildungsverlaufsregister” have been developed and are advanced now (e.g. RatSWD 2022; Schnell 2022). Therefore, even today NEPS is the only data base that allows for in depth study of individual longitudinal competence development and educational trajectories as well as its causes and effects.

Before starting NEPS, there had been intensive discussions about its design and set up, preceded by a review of international and national data on educational processes (c.f. Blossfeld and Schneider 2011). It became apparent that “most empirical evidence in German educational research is based on cross-sectional data like the various international student assessment studies such as Progress in International Reading Literacy Study (PIRLS), Programme for International Student Assessment (PISA), or Third International Mathematics and Science Study (TIMSS)” (Blossfeld and Schneider 2011, p. 36). These studies help to identify trends and to get a picture of the current state of affairs. However, as valuable as—even repeated—cross-sectional studies are, they cannot be used to analyze how distributions or correlations came about, how they will continue to develop, or what the consequences will be. Therefore, longitudinal panel data are needed to describe developments, analyze cause-and-effect relationships over time and identify causal effects.

Besides the argument for a longitudinal design of the NEPS study, a second issue to consider was sampling and thus the analytical perspective of the design. A household sampling strategy, as it is implemented in the Socio-Economic Panel Study (SOEP; Goebel et al. 2019) has many advantages, especially with respect to economic aspects of family wealth and contextual effects. The SOEP, like the later panel studies pairfam (Panel Analysis of Intimate Relationships and Family Dynamics; Huinink et al. 2011) and AID:A (Aufwachsen in Deutschland: Alltagswelten; Rauschenbach and Bien 2012), is a nationwide household panel study that provides data for a wide range of information of all household members, including information on educational issues. However, household compositions change over time. Depending on the design, participants leaving the household either simultaneously drop out of the sample of the respective study (e.g. AID:A) or need to be supplemented with the new (family) members of their new household (e.g. SOEP). With regard to the focus on educational trajectories and individual development, a household design was therefore not considered appropriate.

A cohort panel design, however, is a study design that maps most appropriately individual trajectories and developments and also allows to identify causal effects. An example of this type of study is the British Millennium Cohort Study (MCS). It started in 2001 with newborns (9 months old) as targets and will have implemented seven waves until 2022. By then the target persons are 22 years old and can be found in formal (tertiary) education as well as in the labor market. It took 21 years to collect this data. A cohort study-design allows to closely follow the participant’s biography and can assess ongoing life sequences in a very detailed way. However, as with every panel study, it takes a long time to obtain data on all educational phases of an individual and the information is restricted to one birth cohort only. For this reason, NEPS follows a slightly different design.

## NEPS—a unique endeavor

**Fig. 1:**
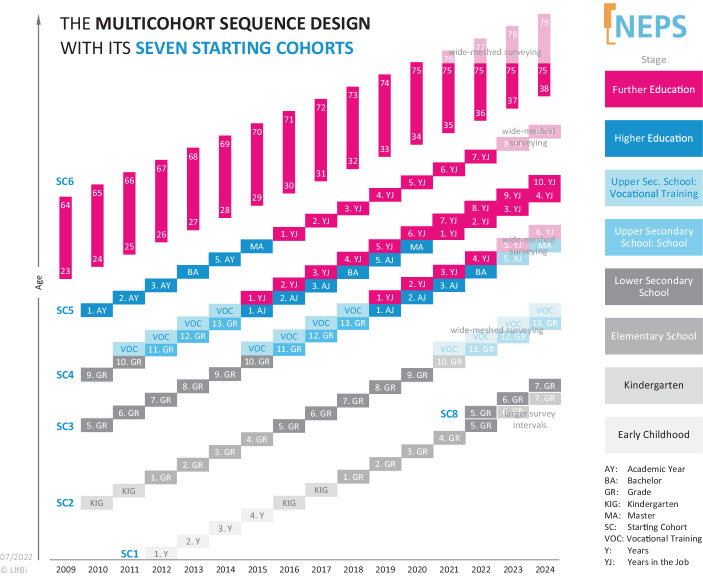
NEPS Multicohort Sequence Design (MCSD) 2009–2024

The main goal of the National Educational Panel Study (NEPS) is to provide a high quality data for research on education from different disciplinary perspectives. The ambitious aim is to study precursors and consequences of education across the lifespan (c.f. Blossfeld et al. 2011; Blossfeld and Roßbach 2019). In order to be able to observe individuals’ competence development as well as the unique passing through different educational stages and sequences, NEPS was designed as a longitudinal study of individuals (target persons). These target persons are surveyed in different stages of the life course, simultaneously taking into account a number of context variables and dispositions. NEPS started with several (mostly) parallel cohorts simultaneously. The main idea of the Multicohort Sequence Design (MCSD, Fig. 1) is to provide information on different stages of education and lifelong learning in a shorter time span and thereby allowing for cross-cohort comparisons. Taken together, six large panel samples were drawn in the first NEPS years and its participants have been followed for more than ten years now. The samples, called starting cohorts (SCs), were drawn and arranged in such a way that each starting cohort (SC) covers different stages of education^2^.

The first data collection started 2009 with the first wave of the NEPS SC6^3^, the adult cohort. The individual sample (first wave) included target persons born between 1944 and 1986 and thus aged between 23 and 64. The vast majority of these SC6 target persons had already left formal education. However, informal and non-formal education do play a major role for adults: In 2020, only the participation rate for non-formal education activities among adults (18–69 year olds) was 57% (e.g. Autor:innengruppe Bildungsberichterstattung 2022). Thus, the corresponding stage within NEPS is referred to as further education. One year after the SC6, the first waves of NEPS SCs 2–5 followed. Based on institutional samples, each of these SCs represents targets in a certain phase of (formal) education: Children (age four) as targets of SC2 were drawn in Kindergarten^4^ two years before school enrollment. Targets in SC3 and SC4 represent two cohorts of students in secondary school: fifth grade students (SC3) after transition into lower secondary school and students in grade nine (SC4) before transition in upper secondary school or vocational track. Targets in SC5 represent first-year students in universities and therefore higher education. Finally, in 2012 the first wave of SC1 data collection took place, again as an individual sample focusing the stage of early childhood with newborns (6–8 months) as target persons.^5^ In 2022 a new cohort of fifth graders was implemented (for more details see Sect. 4.2).

Due to the usually annual surveying of each cohort, target persons are accompanied across several educational stages and various educational transitions. E.g., the target persons of SC4 will have passed through the formal educational system and have entered the labor market in 2022, while target persons of SC2 will have reached the level of upper secondary education. Taken together, the MCSD of NEPS allows to study and compare educational sequences at different points of the educational process and life course simultaneously.

An additional advantage of the MCSD and the overarching and largely comparable assessment framework and survey program (see Sect. 3) is that different cohorts passing through the same educational stages at different times can be compared. E.g. ninth graders being enrolled at different points in time (students of SC4 in 2010, of SC3 in the 2014 and of SC2 in 2020) can be compared with respect to their achievement (assessed with NEPS specific tests) in different competence domains. However, the specific sampling frame of the cohorts (e.g. register sample or institutional samples) affects the robustness of these findings. When comparing fourth graders of SC3 sampled in secondary school and fourth grades of SC1 stemming from a register sample it is not clear to which population the findings can be generalized. However, based on the overarching assessment framework and study program of the NEPS (see Sect. 3) and especially when sampling characteristics are comparable, the MCSD allows to distinguish between age effects (which result from comparing individuals of the same age but in different birth cohorts), cohort effects (which result from belonging to a particular birth cohort), and period effects (which result from a time effect across all age and birth cohorts). This feature applies to SC6 in particular.

Several papers have used the potential of the MCSD by combining data from different SCs. In a recent paper by Skopek and Passaretta (2021) synthetic life courses were built by using target subjects of SC1 (waves 1–5), SC2 (waves 1–6), and SC3 (waves 1–6), thereby covering a period from about half a year to almost sixteen years of age. They compared achievement gaps by parental education across cohorts, thereby focusing on the role of the German education system in reproducing social inequality from birth to adolescents. The results show that achievement gaps emerge and increase long before school enrollment and do not change considerably during the school years. According to the authors’ interpretation schooling prevents existing achievement disparities from widening. There are, however, limitations to the approach of building and interpreting synthetic panels, especially regarding the insufficiently defined population to which the findings refer. Despite these weaknesses the paper takes advantages of the MCSD and points to the potential of the NEPS design for building synthetic panels and for integrative data analysis (IDA, Curran and Hussong 2009).

## NEPS—understanding what is measured

**Fig. 2:**
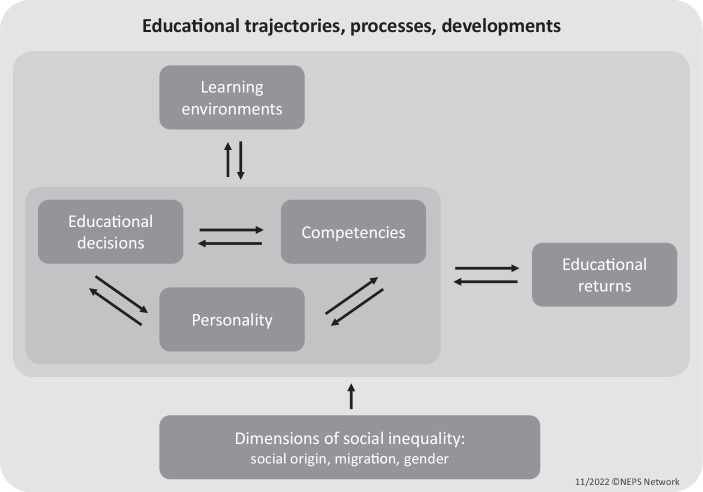
NEPS Framework

The central questions of NEPS are “how competencies unfold over the life course; if and how they influence […] educational careers at various critical transitions; and how and to what extent competencies are influenced in turn by learning opportunities—not only those within the family and the peer group but also those resulting from the way teaching and learning processes are shaped in Kindergarten, school, higher educational, vocational education and training, and adult education” (Blossfeld et al. 2019, pp. 4). At the same time, NEPS aims to describe educational trajectories and decisions and to understand the impact of competencies, contexts, and institutions on these. For its central goal of describing and analyzing individual competence development and educational trajectories over the entire life course, NEPS needs to take into account the role of learning environments and individual (educational) decision-making as well as the target persons’ personality, including traits and motivational, affective, and social dispositions as well as developments. NEPS also implemented an important differential perspective with a focus on social inequality along the dimensions of social origin, migration, and gender. Based on these core themes of relevance that were identified by taking into account relevant theories and research findings, a comprehensive framework was set up by the interdisciplinary NEPS network (see e.g. by Blossfeld et al. 2009). A modified version of the NEPS framework is depicted in Fig. 2. The framework outlines the NEPS’ subject area regarding its theoretical and thematic focal points as well as their interrelations. To this extent, the conceptual framework ensures the coherence of the life-course overarching educational survey and clarifies the analysis perspectives and the central questions and foci.

Taken together the NEPS offers a unique and focused survey program that is characterized below following the ideas of pillars and stages.

### The NEPS pillars—cohort cross-cutting approaches and themes

Six units, the so-called NEPS pillars, bundle together the respective theoretical and analytical perspectives on competence development, learning environment, educational decisions, migration background, returns to education, as well as motivation and personality across the life course (c.f. Blossfeld et al. 2019). The work of the pillars is oriented toward a high quality assessment program in the particular field and is serving NEPS’ overarching (inter)disciplinary research program. They thus ensure a state of the art survey program and enable (in principle) comparability across the eight educational stages.

NEPS has a unique focus on competence development over the life course. Consequently, the main task of the **pillar “Competence Development across the Life Course” **is to develop appropriate test instruments for the assessment of education-related competence domains over the life course. Following the ideas of overarching and comparable measures across cohorts, a framework for each competence domain was set up that ensured that the main competences follow an underlying rational with respect to its theoretical focus and operationalization (c.f. Artelt et al. 2013). The major domains under study are reading, mathematics, and science, which—similar to international Large Scale Assessments (PISA, PIAAC)—are operationalized according to a functional literacy approach. These assessments are supplemented with regular assessments on metacognition, as well as ICT-Literacy—the ability to handle information and communication technologies. Indicators of general cognitive ability will also be included in each educational stage (for a discussion of the conceptual distinction of these measurements see Weinert and Artelt 2019). Within the next years the assessment portfolio will be supplemented in two directions. The assessment of digital competences—ability and knowledge in dealing with every-day digital media use and its influences—on the one hand and declarative general knowledge (c.f. Schroeders et al. 2021) on the other.

In order to be able to describe competence gains and development, competencies measured in NEPS need to be mapped on a common metric for successive survey waves. Therefore, the assessment instruments are developed (based on pilot studies) according to the principles of the respective competence framework (c.f. Artelt et al. 2013) and design features are implemented that allow statistical linkage of the derived person estimate across time (Fischer et al. 2016). The data thus enable a consistent and coherent picture of competencies in each domain (reading, mathematics, and science) across the life course (c.f. Weinert et al. 2019).

There are several studies—also included in this special issue, e.g. *Gnambs and Lockl* (2022)—that focus on competence development in and across different educational and life course related stages. Apart from the papers included in this volume, the following examples illustrate the richness and potential of the data for different research foci. Freund et al. (2021) investigate profiles of individual competence development of (former) SC4 students in mathematics and reading over a period of six years. Using latent growth analysis, they find only one profile, suggesting a general pattern of competence gains in both domains with large gains in mathematics. The authors assume that reading competence favors math proficiency but—opposed to math proficiency—reaches a plateau in secondary education. A second example of a study (Matthewes 2020) on competence development focusses on SC3 students between grade 5 and 7. Matthewes combines NEPS data with data from the nationwide IQB Trends in Student Achievement and thus was able to overcome specific shortcomings in the data sets when studying the effects of early between-school ability tracking on student achievement in mathematics and reading.^6^ His results suggest that early and rigid forms of tracking can impair both equity and efficiency in school systems. An example illustrating the potential of the MCSD and the stage-comprehensive surveying of reading competences is the paper by Locher and Pfost (2020). They investigated the association between time spent reading and reading comprehension throughout the lifespan. The results based on data from NEPS SC3, SC4, SC5, and SC6 indicate that correlations vary considerably over the life course. Whereas medium-sized correlations can be found for leisure-time reading and reading competence for students attending secondary school, the association decreases considerably in adulthood. However, for adults, occupation-related reading predicted reading comprehension.

Another guiding NEPS research question is how individual competence development and educational trajectories influence individual life chances and well-being in the sense of educational outcomes. Corresponding to the competence pillar there is also another, the** pillar “Returns to Education across the Life Course”**, taking care for a stage-comprehensive survey program measuring various returns to education over the life course (c.f. Bela et al. 2018; Gross et al. 2019). NEPS distinguishes between non-monetary returns such as health, satisfaction and social and political participation, and labor market returns such as job quality, working conditions or income. Using NEPS SC6 data, Kamhöfer et al. (2019) 7studied heterogeneity in marginal non-monetary returns to higher education. More precisely, they estimated the effect of college education on cognitive abilities, health, and wages by means of a local instrumental variable technique using exogenous variation in college availability. Overall they found positive effects on a variety of outcome measures (except for mental health), but also substantial effect heterogeneity showing that education does not pay off for everybody. There are also clear signs of selection into gains. “Those individuals who realize the highest returns to education are those who are most ready to take it” (Kamhöfer et al. 2019, p. 228). Complementary to the important substantive findings, the paper illustrates the potential of the large and representative NEPS samples for advanced statistical modelling.

Learning environments are crucial when explaining individual competence development and educational trajectories. Accordingly, the **pillar “Education Processes in Life-Course-specific Learning Environments” **focuses on educational processes in different, interrelated formal, non-formal, informal, and family related learning environments and contexts in which individuals are embedded at particular points in time across the life course (c.f. Bäumer et al. 2019). Clearly, learning environments vary across the life course and are highly heterogeneous. However, the pillar adopts a cross-comprehensive perspective, having developed a structural-processual view and, based on this, capturing analytically selected characteristics of different environments in the NEPS survey program (e.g. child care arrangements, school or adult learning courses). Thus, comparable characteristics oriented towards quantity and quality aspects of the different learning environments (which are added by stages-specific information) are captured on a conceptual level.* The paper by Brosch, Heisig and Zierow (this volume) deals with a comparative analyses of the effects of different learning environments: with data of SC1 they investigate the effect of different forms of early child care (“Tagespflege” vs. “Krippe”) on child development.*

Since the question of the development as well as the causes of social (educational) inequality continues to arise, the NEPS basically adopts a correspondingly differentiated perspective and examines the thematic and theoretical focal points along three dimensions of social inequality, social origin, migration and gender. Therefore, **pillar “Social Inequality and Educational Decisions across the Life Course”** develops and compiles measurement instruments with a focus on sociological explanatory approaches on the extent of and the reasons why educational decisions such as the choice of elementary school, the choice of secondary school, the choice of career, choice of study course, continuation of academic career, or participation in occupational further training vary across different socio-structural groups. (c.f. Stocké et al. 2019). This pillar therefore focuses on class-specific educational aspirations, motivations, expectations of success, and cost estimates, and also pays attention to gender-specific subject choices in educational careers (vocational training, choice of field of study). Zimmermann (2020) made extensive use of the pillars’ constructs by asking: “Social influence or rational choice? Two models and their contribution to explaining class differences in student educational aspirations”^8^. The pillar also provides instruments for recording (highbrow) cultural activities and educationally beneficial resources inherent in social relationships. The latter has also been studied comparatively for adolescents and young adults at different educational stages using NEPS data of SC2, SC3, and SC4 (Hoenig 2019). This paper focuses on normative reference group effects as one and access to resources such as information in the network as another aspect of social capital. In summary, the overall conclusion is that social capital is a fundamental predictor of a successful educational career up to vocational training. A second conclusion is that social capital can explain differences in educational disparities with regard to social origin—with the exception of young adults at the transition to vocational training. Here rather small effects are found and the author attributes this to the fact that the social capital of young people does not distinguish very much at this point.

When looking at social inequality in skill development and educational trajectories, migration background is the most relevant dimension besides social origin. The **pillar “Education Acquisition of Persons with Migration Background**” has developed a broad approach in surveying migration background. In addition to citizenship, migration background is measured in all NEPS SCs by the country of origin back to the third generation of the target persons (Kristen et al. 2016). *This unique feature is applied in a paper by Hunkler and Schotte (this volume) by addressing academic achievement and psychological adaption of 3rd generation students in Germany*. The migration pillar also emphasizes capturing migration-specific characteristics that are relevant for educational integration over the life course (c.f. Kristen et al. 2019). One focus lies e.g. on the assessment of students’ knowledge and competencies in the language of their parents’ country of origin. Edele and Stanat (2016) nicely illustrate theoretically driven research on language development using this NEPS feature. Based on SC4 data they tested and confirmed the hypothesis that listening comprehension in L1 [first language] is a significant predictor of language minority students’ reading comprehension in L2 [second language] using Russian and Turkish as language of origin. Given the varying language similarity of the two languages to the German language (L2) the transfer findings seem to be language-independent. Furthermore and jointly with the pillar on social inequality and educational decisions, the migration pillar also focusses on migration-specific aspects of social and cultural capital or learning environments. Currently the program is being supplemented by surveying different facets of (perceived) ethnic discrimination which aims to shed light on the question of the role of ethnic discrimination in explaining ethnic inequalities (Horr et al. 2020).

Last but not least NEPS takes into account different facets of target persons’ personality, also including motivation and socio-emotional skills. These variables serve as both, as predictors and as outcomes of education. Additionally, they are regarded as key for understanding competence development and educational decision-making. The sixth **pillar “Motivation and Personality across the Life Course”** thus contributes constructs on personality and motivation, including interest (e.g. learning motivation, subject related and general interest), self-related cognitions (e.g. school-related/academic self-concept of students in school and higher education institutions, self-esteem), and indicators of social competencies across the life course. Furthermore, the Big Five personality dimensions are covered (c.f. Wohlkinger et al. 2019). An example of research using constructs from this pillar is the paper by Heyder and Brunner (2018).^9^ It examines the relationship between student achievement and helplessness and the moderating role of teachers’ belief that talent leads to performance. Using NEPS SC4 they find that teachers’ beliefs reinforce the achievement-helplessness link in the school subject mathematics but not in German.

Complementary to the six pillars, “**NEPS sociodemographic basic information**” ensures that sociodemographic information is collected in a comparable way across the NEPS stages and SCs. Among others, the variables gender, age, citizenship, and migration background are covered. The unit also makes sure that central information for the indicators of education and employment such as International Standard Classification of Education (ISCED), Comparative Analysis of Social Mobility in Industrial Nations (CASMIN), “Klassifikation der Berufe (KLdB)”, or International Socio-economic Index of Occupational Status (ISEI) are assessed.

In addition, a highly coordinated **life course survey program** was developed for (young) adults (SC3-6) containing structural information about educational participation and life course situations and circumstances (c.f. Anger et al. 2019). In several modules, successive questions about different areas of life are asked. It is thus possible to generate retrospective information on an individual life course that consists school attendance, vocational preparation, and training and participation in higher education. In addition, labor market participation and phases of unemployment are recorded as well as information on partnerships, children, and parental leave. These questions are complemented by stage-specific modules, e.g. on retirement for target persons older than 55 years of age. In each year of the survey the current status of each target person is updated via dependent interviewing. The module on school attendance is also applied in the lower SCs where information on school participation (as well as on childcare arrangements) is collected and updated in interviews with parents.

### The NEPS stages—stage specific approaches and themes

Complementary to the overarching themes of the NEPS pillars, NEPS stages focus on specific stages of education and life course. Each of the eight NEPS stages (see Fig. 1) integrates the relevant research into the respective stage-specific survey program in order to be able to provide data to answer current research questions related to each stage. To this end, (a) constructs are derived from stage-specific theoretical approaches and (b) (new) measurement instruments are developed or compiled to complement the instruments of the pillars.

In the youngest NEPS stage “**Newborns and Early Childhood Education**” (stage 1) central questions relate to the adequate assess of competencies of newborns and infants in their first years up to four years of age. These are needed for the study of competence development in the early years, the prediction of later competences as well as major influential factors (c.f. Hachul et al. 2019). Consequently, this NEPS stage provides early competence measures based on observations of the target persons at home, e.g. measurements of the so-called habituation-dishabituation paradigm. Furthermore, the stage team developed an instrument to gather information on aspects of mother-child-interaction and shed a specific light on the family as well as parents’ needs and orientations. Burghardt et al. (2020) addressed the question of the relevance of the early childhood home and institutional learning environment at the age of two years, on mathematical competencies at the age of four, using data from SC1. Their research shows positive effects of parent-child interactions in the early years on later mathematical competencies.

In the next two stages, “From Kindergarten to Elementary School” (stage 2) and “From Elementary School to Lower Secondary School” (stage 3), the main focus lies on effects of early childhood education and care (ECEC) and early institutional education on competence development and educational trajectories (c.f. Berendes et al. 2019). A further focus is on the relation of ECEC and informal learning environments especially in the family as well as their role in closing achievement gaps and social disparities (social origin but also migration) in the development of preschool children. Instruments are developed and data provided to investigate mediators within and between children’s (pre)school learning environments. In these stages the assessments of competencies focus on early scientific and mathematical literacy as well as language competencies (e.g., vocabulary, grammar, phonological awareness). Furthermore, parents and educators are asked about their perception of the target children’s skills. The development of language competencies received special attention in the stage’s survey program, e.g. by surveying information on support for language acquisition in ECEC institutions (also for natives, not only for migrants) and at home. *Möwisch et al. (*2022*) (this volume) studied vocabulary development of mono- and multilingual German children and asked whether domain-specific maternal interaction behavior at the age of two has an impact on vocabulary development of mono- and multilingual German children in the age of three to seven.*—*Olczyk et al. (*2022*) (this volume) investigate social and gender bias in teacher assessments of students’ language and mathematical skills at school entry, in grade 1, and additionally examine the effect of (potentially biased) teacher assessments measured in grade 1 on student achievement in grades 4–5 resp. at age 10–11. As this paper additionally provides a comparison across three countries and datasets (Germany/NEPS SC2; UK/ MCS; US/ ECLS-K) it also shows the potential of NEPS for international comparisons.* The potential of the NEPS for international comparative research with policy impact is also shown in an analysis of socioeconomic status differences in children’s cognitive development in Germany and the United States by Linberg et al. (2019). The authors can confirm country differences, e.g. regarding parents’ educational status. They also find differential effects with respect to the factors explaining social disparities in children’s cognitive development: in US the families’ financial resources and poverty are important drivers explaining the gaps while in Germany the factor migration background plays a major role.

Regarding the transition from primary to secondary education the NEPS survey program offers detailed information about the related recommendation. Bittmann (2021a) used these school recommendation information by studying the long term effects of parents not acting according to these recommendation. Using SC3 data he reports negative long-term consequences regarding competence development, well-being, and certificates which can be explained by a mismatch between the demands of the school type and students’ academic performance. He found no consequences for health or absenteeism. This paper again shows the high potentials of the NEPS investigating long-term effects of decisions on educational trajectories and sheds light on the effect of transition regulation.

The next stage “**From Lower to Upper Secondary Education**” (stage 4) focuses inter alia on the extent to which students’ competencies correspond to the type of school they choose or are recommended to attend and how their competencies develop depending on the type of school they attend (c.f. Fabian et al. 2019). In addition, data are collected to investigate questions about the frequency and causes of school type changes or class repetitions and their impact on further educational progression and success. Class repetitions can be seen as a critical event in life, which has negative impact on children’s well-being, as Vockert et al. (2019) show with data of SC3. In addition, the focus of stage 4 in the first NEPS period was on capturing the development and importance of orthography as a core secondary school competency and corresponding beneficial activities such as reading as well as attitudes to them (c.f. Jarsinski et al. 2016). Preparing for the new cohort of fifth graders (see Sect. 4.2) one of the new foci of the stage is on developing tests and instruments measuring the academic promotion, acquisition, and development of civic literacy.

If the individual’s educational path continues in the general education school system, the student passes “**Upper Gymnasium Level and Transition to Higher Education, Vocational Training, or the Labor Market**”, that is treated in NEPS stage 5 (c.f. Wagner et al. 2019). In upper secondary education it is intended to impart general education (in addition to in-depth specialized knowledge) and prepare students for scientific studies (scientific propaedeutics). By awarding the Abitur, it is also intended in some way to ensure that the young adults are suitable for university studies as the Abitur is the formal admission qualification.^10^ If the individual’s educational path continues not in the general education school system, there are several situations that should be taken into account. It is the focus of stage 6 to examine the transitions of adolescents and young adults “**From Vocational Training to the Labor Market**” (c.f. Ludwig-Mayerhofer et al. 2019). The questions in stage 6 concern the access to the vocational training system, passing through one or more training measures (also dropout) and finally entering employment. Furthermore, the question is how these different status passages influence each other and which factors particularly affect the respective status and transition. The stage concentrates on the development of measurement instruments for vocational orientation, a topic relevant to all students, also those at the end of upper secondary school. A special focus of the stage is on young people leaving school without any certificate. Therefore the basic program surveying life histories is additionally gathering information on vocational preparation developed by the stage. *Holtmann and Solga (this volume) use at least this information and the rich data of SC4 to analyse the black box of dropping out of vocational training. *There is also a publication by Hillmert et al. (2017) on the study of transitions to vocational training that not only provides interesting results but also shows that NEPS data can also be enriched by external, regionally aggregated data.^11^ Based on SC6 data and retrospective life course data about vocational training, they study the influence of differences and changes at the local labor market on school leavers’ transition behavior into vocational training. Based on a decomposition method distinguishing effects of long-term change, short-term fluctuations, and regional structural differences of the labor markets, they were able to show significant effects of the long-term development of the regional labor market: especially low-qualified school leavers had difficulties to find training positions in the dual system in case of a negative long-term trend of the regional unemployment rate. For short-term fluctuations the effects were rather small. The authors conclude that the decomposition of the differences and changes in structural variables is important to adequately measure the effect of context variables.

The last stage of the German education system is treated in the stage 7 “**From Higher Education to the Labor Market**” (c.f. Brachem et al. 2019). Since individual pathways through tertiary education are regularly characterized by two parts (bachelor degree and master degree), central questions in this stage address mechanisms behind (1) these two educational decisions, (2) choices or changes of subjects of study, and (3) (complete) drop out as well as the question of social inequality in all three foci. To investigate the questions in detail, the stage developed instruments (in addition to the theoretical concepts of the decision pillar) derived from the theoretical model of the expectancy-value theory (c.f. v. Keyserlingk et al. 2021) as well as from Tintos’ concept of academic and social integration (for an analysis c.f. Schaeper 2020). Ochsenfeld (2016)^12^ investigated how the persistence of horizontal sex segregation in college majors can be explained. With a discrete choice design and SC5 data the author showed that differences in mathematics achievement fail to explain gendered patterns of selection into college majors. Additionally he found out that internalized vocational interests can explain the gender specific choice better than an orientation at expectations of parents or peers and that it cannot be explained by women’s anticipation of gendered family roles nor from their anticipation of sex-based discrimination. Another open question in this stage concerns the non-traditional university students, their way through higher education, and their returns to education compared to traditionally qualified university students as there is known little about this group. Due to the survey program introduced above relevant information is gathered to investigate this question. *Ordemann* (2023)* is dealing with this question with data of SC5 linked to administrative data from the Institute for Employment Research (IAB).*

As NEPS is focusing competence development and educational trajectories over the life course, educational processes are also observed in adult age. In stage 8, “**Adult Education and Lifelong Learning**” especially the question about participation in further education is addressed (c.f. Allmendinger et al. 2019). A very broad definition of continuing education is taken as a basis, and professional continuing education after the first training (“Erstausbildung”) is considered just as much as privately motivated continuing education, once again in a wide variety of settings—formal, informal, and non-formal (e.g. courses but also participation in conferences). The open questions driving the new survey program of stage 8 are concerned with the consequences of the technological change and digitalization of the labor market for participating in (further) education, competence development in adult age, and educational returns for different birth cohorts. To be able to investigate these consequences the stage developed instruments e.g. to access changing job tasks and the level of digitalization of tasks, triggered demand for further education, and the perceived consequences. *Further education in adulthood is also addressed by Kohl and Martin* (2023)*. They investigate the effect of further education on health behavior, looking on subgroups, e.g. older people of SC6. Additionally Kleinert and Zoch* (2023)* study (occupational) learning in adulthood and the consequences of the Corona-Pandemic on unequal learning chances with data of SC4, SC5 and SC6 (see some detailed information on the additional data on the Corona-Pandemic next)*.

### Adding additional content to the NEPS

The global spread of the SARS CoV‑2 virus has been affecting all areas of daily life since 2020. It also raised a wide range of research questions related to education, for which longitudinal data, such as those from NEPS, are particularly relevant. Against the background of the core topics of the NEPS—competence development and educational trajectories over the life course—the following questions in particular arise: How are learning environments changing and what potentials and risks are becoming apparent as a result of the incipient digitization of learning? Are there effects on upcoming educational decisions (transition decisions/participation) and are there medium- and long-term effects of the Corona pandemic on educational outcomes, such as income, but also non-monetary returns, e.g., health/health behavior, labor market participation?

To investigate these questions and the medium- and long-term effects of the Corona pandemic on competence development and educational trajectories over the life course, not only longitudinal data on the core topics above have to be available but also direct consequences of the Corona pandemic in the areas of daily school, work, and family life (e.g. the extent of homeschooling and/or home working during lock down and the perceived burden). Therefore a concept was developed and these specific Corona questions were collected in the so-called **NEPS supplementary surveys on the Corona pandemic (NEPS-C)**. This will allow us to explore both retrospective differences in responses to the crisis and long-term consequences for educational biographies in Germany. In order to collect these data in a timely manner, the first questions were administered via online surveys in the NEPS SCs 2–6 in May 2020, together with a panel maintenance measure. These data are already available to the scientific community. Furthermore, the integration of the corresponding questions in the so-called NEPS additional module on the corona pandemic has been realized for the main surveys in all SCs. These additional NEPS‑C data are and will be made available to the research community after careful preparation and anonymization at the latest as part of the regular release of the Scientific Use Files.

In order to cope with the actuality of the topic and to be able to provide at first descriptive results promptly, the NEPS network and especially a team of NEPS staff members at LIfBi were working on first analyses in advance. Insights into research results are provided in the transfer series “NEPS Corona und Bildung”, a paper series addressed to the wider public (e.g. Handschuh et al. 2021; Wolter et al. 2020; Zoch et al. 2020). Further analysis are already published in books or journals (e.g. Bittmann 2021b; Nusser 2021; Zoch et al. 2021). *Also in this volume a paper by Lorenz et al.* 2023*can be found working on the question about effects of the social context on dealing with homeschooling.*

In addition, NEPS network is striving to open up the survey program to external content. For the first time, an **open “Call for Modules” (CfM)** was launched in 2020. Proposals for question modules in all SCs and for all target groups for surveys starting in fall 2021 could be submitted. Modules were allowed to be up to 5 items in length. Proposals for methodological questions were also possible. After a two-stage selection process with a scientific review procedure, in which the LIfBi Scientific Advisory Board was also involved, three proposals were selected. The new modules “Self-efficacy for motivational regulation” in SC1 , “The innovative teacher—proactive behavior in the profession” in SC5 and “Conspiracy mentality and political deprivation” for several SCs now complement the NEPS survey program with content that is likely to be highly relevant for questions from different scientific disciplines. The second **NEPS-CfM 2022 **was launched in July 2022. This thematically narrowed call is intended to capture additional target dimensions of education or dimensions that have not yet been adequately considered in the NEPS (e.g., in the area of general skills and abilities or attitudes). The proposals for quality-tested questions/items with a validated duration of up to two survey minutes in the telephone interview are to be implemented in the so-called outcome CATI for the NEPS SCs in the wide-meshed surveying (see Sect. 4.1). It is planned to open the NEPS survey program to external content on a regular basis.

## NEPS—lines of future development

After a survey period of more than 10 years, panel participation rates are very stable at a high level in all SCs (see data documentation for each SC at www.neps-data.de; for an eight year perspective see Zinn et al. 2020). NEPS has thus achieved its goal of providing parallel data for describing and analyzing individual competence development and educational trajectories over the entire life course: comparable data are available from about 60,000 target individuals, some of whom have passed through several educational stages. Even the youngest NEPS participants have now reached secondary school, and the former students from SC3 or SC4 and students from SC5 now grown up have largely completed their initial vocational training and entered working life. Thus, many of the NEPS participants are now in a comparable educational situation (further education), so that the claim of the MCSD to provide data from different cohorts in the same educational stages can no longer be fulfilled. It should also be noted that selectivity according to social origin and migration background has also emerged in the NEPS SCs, which raises the question of the extent to which further close monitoring of all SCs continues to appear useful. In response to these two trends, the decisions outlined below were made with an eye toward key lines of NEPS development and further planning.

### Concept of wide-meshed surveying (WMS)

As shown in Fig. 1, the so-called wide-meshed surveying starts 2021 for participants of SC6 older than 75 years of age and SC2, in 2023 for SC3, and SC5 as well as 2025 for SC4 (SC1 still has to be planned). Wide-meshed surveying (WMS) indicates the change of the surveying concept from an annual, close and detailed monitoring to a more wide-meshed and shorter one. In addition to panel maintenance and tracking measures, the concept includes, in particular, the survey of key educational outcomes among panel participants, usually every five years. In the future, the cohorts in the WMS will be explicitly opened for add-on studies by external researchers. Currently, different models and types of add-on studies are being considered and a corresponding process is being discussed and agreed upon so that the procedure of approval is as transparent as possible and the respondents in the WMS cohorts are contacted in a balanced manner.

### New cohort of fifth grades: starting cohort 8 starting in 2022

As can also be seen from Fig. 1, a cohort redrawing in the lower secondary school context, a new cohort of fifth graders (SC8) started in 2022.^13^ This means that twelve years after the start of the first school cohort of fifth graders, a second school cohort was established in order to be able to map the effects various changes of the last decade, including social and educational policies. These changes are, for example: changes related to school structure (e.g., development of school system toward a two-tier system, return to 9‑year Gymnasium, increase of private schools and schools offering university entrance certificate as formal degree), increasing heterogeneity in schools due to structural changes in society (inclusion, refugees, and new immigrants) as well as technological change and digital learning (that is an important perspective for the whole NEPS survey program).

On the one hand, comparability between the new (2022) and the old (2010) cohort of fifth graders is strongly emphasized. On the other hand, there are innovative survey methods, new content foci, and additional competence measures, e.g. extending the portfolio of cognitive competencies by digital competences and civic literacy. The aim is to record developments in different areas of competence as well as educational processes, decisions, and outcomes and to link them to a number of contextual factors (including institutional and familial) and conditions during the secondary school years. The design includes surveying students in the classroom-context (with an improved sampling concept to be able to gather more information on the class level), teachers and head of schools as well as parents. Several survey modes are used to address the different target groups most effectively.

Another unique feature of the new SC8 is the implementation of a school panel, focusing on school development. For this sake, not only the teachers of the target persons and the school head are interviewed but the whole teaching staff of a school. The questions address teacher cooperation, school culture and educators’ own further education. In addition, the school panel is also a part of the (planned) twofold combination with IQB Trends in Student Achievement^14^ by (1) implementing overlapping school samples of IQB Trends 2022 and SC8. Furthermore, (2) IQB Trend tests (reading & mathematics, end of elementary school) are implemented in SC8’s grade five assessment. The design enables to address issues related to the short and long-term impact of students’ educational standard proficiency levels (e.g. minimum vs. optimum standards) at the end of elementary school on the further pathways through secondary school and beyond.

### Further cohort succession

In addition to the developments described above, a second cohort of newborns/children in early education is being planned (SC7). The aim of this cohort succession is to be able to examine the effects of social and educational policy changes that may emerge in the area of early childhood education, particularly as a result of the expansion of the childcare system and related political regulations. Therefore, the planning for the new SC7 will also focus on a high degree of content comparability not only, but especially, to the first cohort of newborns (SC1) for the corresponding relevant questions. In addition, against the background of technological change, new focal points in this respect and further focal points regarding content are also discussed, as well as the use of innovative survey methods.

Furthermore, the sample of the existing cohort of adults (SC6) will be refreshed and enhanced in order to be able to continue to use these data to conduct research on the effects of technological and demographic change, but also of the ongoing educational expansion, on educational and learning processes and their returns to education. However, SC6 increasingly lacks younger birth cohorts in the sample, which need to be enhanced in order to be able to make statements about younger age groups as well. Given that individual birth cohorts’ sample size is thinning, a refreshment concept for respective birth cohorts is currently developed. Representation of all birth cohorts in the adult sample is important to distinguish cohorts, periods, and age effects.

In summary, NEPS, which has now been running for more than 10 years, provides comprehensive data on the educational trajectories over the entire life course of about 40,000 individuals across Germany. NEPS data will be enriched soon by the second cohort of fifth graders, by a refreshment and enhancement of the adult cohort in 2025, and in the medium term by another cohort of newborns/children in early education. In addition to these innovations in the MCSD, conceptual consolidations for the NEPS survey program are also planned, which will incorporate not least the needs of the scientific community of the NEPS users (e.g. surveyed in the NEPS user survey). Thereby, the longitudinal assessment of competence development and educational trajectories over the life course as well as the panel measurement of their preconditions and results, remain central for the conceptualization of the NEPS survey program and design to enable further research on their dynamic and reciprocal interactions.

## Footnotes

1 Blossfeld served as the PI of the NEPS until mid-2012. He handed over to H.-G. Roßbach and since 2014 the National Educational Panel Study has been conducted by the Leibniz Institute for Educational Trajectories (LIfBi) and is headed by its director. Today, the network consists of 30 network partners and their teams, spread across 13 research institutions and universities in Germany.

2 For an overview on sampling procedures, see Aßmann et al. (2019). For information regarding panel development, see data documentation for each SC on www.neps-data.de.

3 NEPS was able to take on the ALWA study (Kleinert et al. 2011) of the Institute for Employment Research (IAB) in Nuremberg and integrate the sample into the NEPS SC6 and also use parts of the surveying instruments. Consequently, this SC could start one year earlier than the other SCs.

4 In Germany “Kindergarten” is a type of daycare for children who are under 6 years old. It is mostly organized in mixed age groups without (as e.g. in the US) having a standardized set curriculum. Nevertheless, Kindergarten has an educational mission and in the last year before school enrollment there is usually a certain amount of time in preschool classes preparing explicitly for school (Kultusministerkonferenz 2022).

5 The start of SC1 was recommended by the reviewers during the application process. Accordingly, the preparation could also start later and as a result the first field started two years later than SCs 2–5.

6 The paper was awarded the NEPS Publication Award 2020.

7 The paper was awarded the NEPS Publication Award 2018.

8 The paper was awarded the NEPS Publication Award 2019.

9 The paper was awarded the NEPS Publication Award 2018.

10 Another question in this stage in the first years of NEPS was the impact of structural reforms in the “gymnasiale Oberstufe”, in particular the investigation of effects shortening the gymnasium period from 9–8 years on students’ academic performance and motivation, or the abolition of traditional basic and advanced courses. Therefore two additional studies were conducted in NEPS that have been completed meanwhile. The data are available as Scientific Use Files (SUF) at the neps data center. For central results based on these data see Hübner et al. (2020). The paper was awarded the NEPS Publication Award 2019.

11 The paper was awarded the NEPS Publication Award 2017.

12 The paper was awarded the NEPS Publication Award 2016.

13 The numbering of the new cohort of fifth graders with SC8 takes into account that the planning of the cohort succession further foresees the drawing of a new cohort of newborns, designated as SC7, even if it will be implemented chronologically later (see Sect. 4.3).

14 IQB Trends are designed for monitoring the extent to which German students achieve the national educational standards (binding in all federal states, see https://www.iqb.hu-berlin.de/bt). There are assessments at the primary level [German (reading, listening comprehension) and mathematics] carried out every five years since 2009. The latest assesment took place in 2021 instead of 2020 due to Corona. Every three years there is a changing assessment at secondary level I [German/English/French or mathematics/science]. In the next wave 2022 German, English, and in part French will be surveyed.
